# Optimizing sampling rate of wrist-worn optical sensors for physiologic monitoring

**DOI:** 10.1017/cts.2020.526

**Published:** 2020-08-25

**Authors:** Brinnae Bent, Jessilyn P. Dunn

**Affiliations:** 1 Department of Biomedical Engineering, Duke University, Durham, NC, USA; 2 Department of Bioinformatics and Biostatistics, Duke University, Durham, NC, USA

**Keywords:** Biomedical informatics, wearable sensors, health information management, big data applications, data compression, data engineering, data handling, data integration

## Abstract

**Introduction:**

Personalized medicine has exposed wearable sensors as new sources of biomedical data which are expected to accrue annual data storage costs of approximately $7.2 trillion by 2020 (>2000 exabytes). To improve the usability of wearable devices in healthcare, it is necessary to determine the minimum amount of data needed for accurate health assessment.

**Methods:**

Here, we present a generalizable optimization framework for determining the minimum necessary sampling rate for wearable sensors and apply our method to determine optimal optical blood volume pulse sampling rate. We implement *t*-tests, Bland–Altman analysis, and regression-based visualizations to identify optimal sampling rates of wrist-worn optical sensors.

**Results:**

We determine the optimal sampling rate of wrist-worn optical sensors for heart rate and heart rate variability monitoring to be 21–64 Hz, depending on the metric.

**Conclusions:**

Determining the optimal sampling rate allows us to compress biomedical data and reduce storage needs and financial costs. We have used optical heart rate sensors as a case study for the connection between data volumes and resource requirements to develop methodology for determining the optimal sampling rate for clinical relevance that minimizes resource utilization. This methodology is extensible to other wearable sensors.

## Introduction

The practice of personalized medicine, including the combination of clinical, genomics, imaging, wearables, and “real-world” data, has the potential to revolutionize healthcare. However, this rapidly growing digital health trace poses significant challenges in healthcare data management [1]. By 2020, the total amount of digital healthcare data worldwide is projected to exceed 2000 exabytes (equivalent to 2 trillion GB) [2]. Healthcare data storage requirements are quadrupling every 2–3 years and are projected to cost up to $600 billion/month by 2020 [3,4]. The growing need for data storage and compute power is driving a move toward secure cloud computing [1,5]. Currently, up to 80% of health data collected in clinics is considered unusable because it is spread across numerous repositories and cannot be easily linked to the electronic health record (EHR) [3]. In addition to expanding medical storage capabilities, efficiency, usability, and compute power, we must determine how to trim data volumes appropriately to retain important information while removing unnecessary or repetitive information.

Sampling rate refers to the rate, or frequency, at which data are collected per second. For continuous monitoring of the electrical activity of the heart and heart rate variability (HRV) using the electrocardiogram (ECG), data are typically sampled at 1000 Hz which requires approximately 192 kB/second of data storage [6]. Because of the extensive storage requirements of continuous data and challenges surrounding its interpretability, the current data from continuous monitors like ECG are only stored in the EHR as summaries of the raw (signal-level) data. However, it would be useful to preserve this raw data for research and clinical applications that include the development of algorithms and digital biomarkers for improved patient care and understanding of physiology.

Monitoring of vital signs has traditionally been limited to clinical visits with few exceptions. This provides a very small window into a patient’s daily health and wellness. Continuous monitoring using wearable sensors provides a more comprehensive view of a patient. While wearable sensors generally sample at a much lower rate than ECG to preserve battery life, they also provide longitudinal data, which could lead to a data deluge. Determining the minimum sampling rates required for wearable sensors to be relevant for clinical and research use will enable use of this data within the clinical research ecosystem. Another motivation to decrease sampling rate is the trade-off between battery power consumption and sampling rate. Higher sampling rates have increased power consumption, which decreases battery life [7–10].

One wearable sensor that is used regularly in both consumer and clinical grade wearables is photoplethysmography (PPG), a cost-effective, noninvasive optical technique for measuring blood volume changes which result from the mechanics of pulsatile flow of the cardiovascular system. A standard PPG device is composed of a light source (typically a light-emitting diode [LED]) and a photodetector. There are two types of PPG: transmittance- and reflectance-based PPG. In transmittance-based PPG, tissue is between the LED and the photodetector (e.g., on the finger probe or ear clip). Reflectance-based PPG reflects light into tissue and the light reflected is collected with a photodiode that is located next to the LED (e.g., wrist or forehead PPG). Heart rate and approximate interbeat intervals (IBI) can be extracted from the PPG signal using peak detection methods. PPG is the technology behind most continuous wrist-based heart rate monitors on the market today, and this wide use makes PPG an optimal technology for exploring clinically relevant diagnostics. Wearable wrist-worn PPG is popular due to its portability, affordability, and ease of user experience with a seamless transition from watch to wearable.

HRV is a clinically relevant marker that can be derived from PPG [11] and is a widely used metric to evaluate autonomic nervous system function. HRV is the fluctuation in the time intervals between adjacent heartbeats and low HRV is linked to numerous chronic conditions, including diabetes, hypertension, cardiovascular disease, and psychological illness [12,13]. Traditional metrics of HRV are measured using electrocardiography (ECG), which records the electrical activity of the heart. However, ECG is traditionally limited to clinical assessment or short-term home monitoring because it is inconvenient and costly. Wearable PPG monitoring of HRV is a more convenient and continuous solution as compared to ECG, but it generates even larger volumes of data due to its longitudinal nature.

ECG and finger probe PPG sampling rates have been thoroughly examined to minimize data volumes required for HRV diagnostics [14,15]. While the optimal sampling rate for transmittance-based PPG on finger probes has been explored in silico [16], no study has yet examined the reflectance-based PPG that is utilized in wrist-wearable sensors against ECG to determine the optimal sampling rate. *To our knowledge, no study has examined the minimum sampling rate of wrist-wearable PPG for both HR and HRV diagnostics.* The purpose of this study is to examine the limitations of wrist PPG for determining the minimum sampling frequency necessary to obtain clinically relevant HRV metrics when compared to the gold standard ECG.

## Methods

### Optimization Framework

**Fig. 1.**
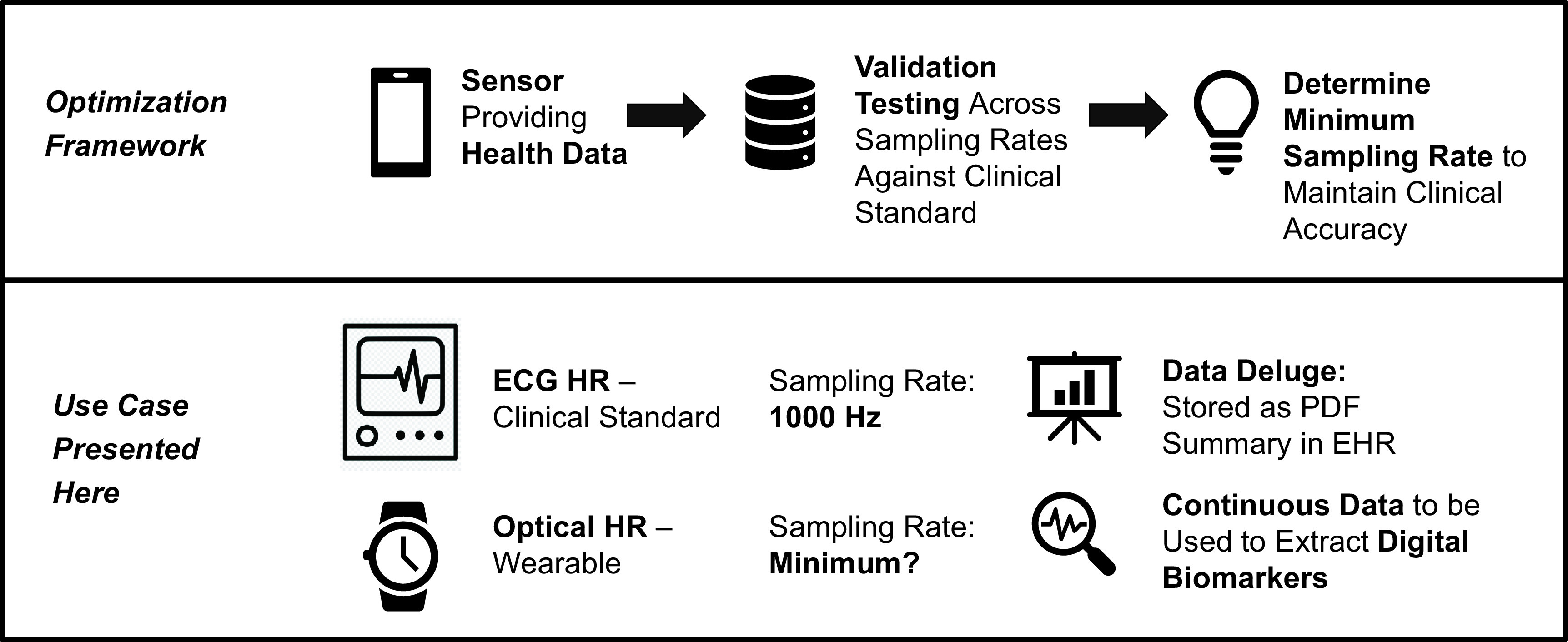
Sampling rate optimization framework. The optimization framework presented here takes a sensor providing health data and performs validation testing across sampling rates against the clinical standard in order to inform minimum sampling rate to maintain clinical accuracy. The use case we present here uses optical HR measurements (photoplethysmography, PPG) from wearable devices. This PPG data is compared to ECG, the clinical standard, across the different PPG sampling rates. The goal is to use continuous PPG measurements to continuously extract digital biomarkers to report in the EHR rather than a single timepoint ECG which results in a single summary stored as a PDF.

The optimization framework presented here examines a sensor providing health data and performs validation testing across sampling rates against the clinical standard in order to inform minimum sampling rate to maintain clinical accuracy (Fig. 1). We then apply this framework to optical HR (PPG) measurements on wearable devices. This data is compared to ECG, the clinical standard, across sampling rates.

### Data Collection

This study is a retrospective analysis of a previously conducted study [17]. A total of 56 participants were recruited for the original study. Data from three participants were excluded from the original study due to incomplete ECG records. Of the remaining 53 participants, 16 participants had either missing (10 participants) or incomplete (6 participants) IBI data. Thus, we utilized the remaining 37 participants for this analysis. We only utilized the data when the participants were seated at rest. Five minutes, the standard for short-term HRV analysis [12], was chosen as the time duration that HR and HRV metrics were calculated over for this study.

ECG and PPG were recorded simultaneously for 5 min from 37 volunteers (22 females, 25.4 ± 6.0 years, 169.8 ± 9.1 cm height, and 64.3 ± 10.8 kg weight. All Fitzpatrick skin tone categories were represented). Data were collected while participants were at rest in an upright seated position. PPG data were recorded at 64 Hz (the standard sampling rate of the device) from the right wrist using the Empatica E4 wristband. ECG data were recorded at 1000 Hz from three leads using a Bittium Faros 180 ECG.

**Fig. 2.**
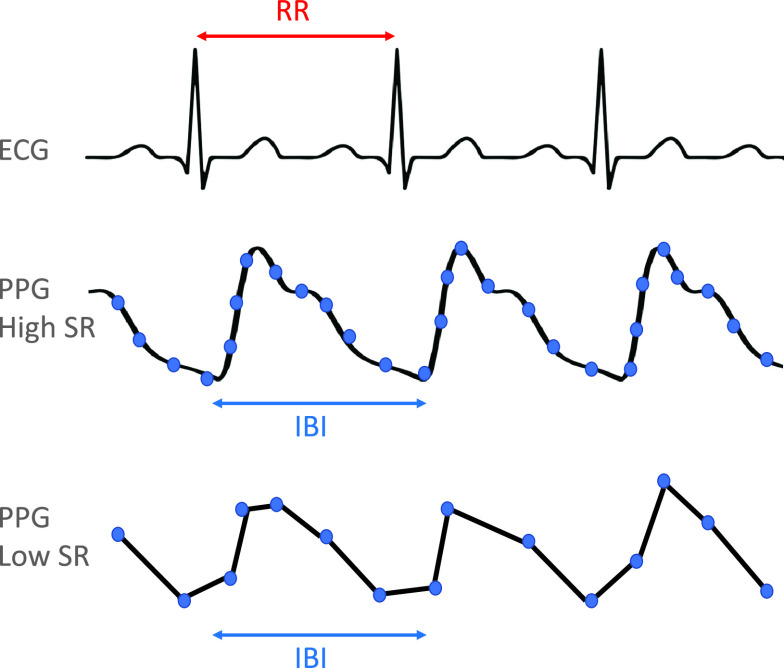
Comparison of ECG (RR intervals) and PPG (IBI). Decimation reduces the number of points (shown in blue) by an integer factor. ECG is shown sampled at 1000 Hz. PPG sampled at a high sampling rate (i.e., 64 Hz) and PPG sampled at a low SR (i.e., 16 Hz) are shown.

Raw ECG was processed using the clinical standard, Kubios HRV Premium (version 3.3) to extract RR intervals, as shown in Fig. 2. PPG data from the Empatica E4 device are supplied as both raw PPG (green LED light only) and an IBI sequence. The IBI sequence provided by Empatica is obtained from their wristband-integrated processing algorithm that removes peaks deemed likely to be incorrect due to noise in the raw PPG signal, which they compute from the red and green LEDs on the device. Red LED PPG signal is not provided and, to our knowledge, is only used in the calculation of the provided IBI sequence. Because we do not have access to the raw red LED data from the device, we utilized the IBI sequence provided by Empatica to reverse engineer the signal and remove motion artifacts that were removed by the Empatica red LED-based algorithm.

Our updated PPG signal could then be downsampled. In order to examine the sampling frequency effect on HRV metrics, we generated the downsampled PPGs by decimation, which divides the sampling rate by an integer factor after low-pass filtering the signal. First, high-frequency signal components were reduced using a standard digital low-pass filter (IIR, Chebyshev order 8) to reduce distortion from aliasing [18]. Next, decimation via rate reduction by an integer factor was performed. The PPG signal (original 64 Hz) was decimated into frequencies by factors of 64: 64/1, 64/2, 64/3, 64/4, 64/5, 64/6, 64/7, 64/8, 64/9, and 64/10 Hz. A Kolmogorev–Zurbenko low-pass linear filter [19,20] and outlier removal were used to mitigate any additional motion artifact not removed by the Empatica processing algorithm. Following the process described by Empatica for determining their IBI sequence, local minima were detected using a rolling minimum detector and the IBI values were calculated as the difference between these local minima values, as shown in Fig. 2. Outlier capping at 1.5 * inter-quartile range (IQR) was performed for each downsampled signal. The preprocessing pipeline code is available here: https://github.com/Big-Ideas-Lab/OptimizingWearableSR.

### HRV Metric Calculation and Statistical Analysis

We examined HR (mean HR, minimum HR, maximum HR) from the ECG and PPG. We analyzed HRV from the ECG and PPG using standard HRV metrics in the time domain: HRV (mean HRV, median HRV, maximum HRV, and minimum HRV), SDNN (standard deviation of the NN interval, which corresponds to all of the cyclic components responsible for variability in the period of the recording because variance is equal to the total power of the spectral analysis), RMSSD (square root of the mean squared differences of successive NN intervals; reflective of the beat-to-beat variance in HR and used to estimate the vagally mediated changes in HRV [12]), and pNN50 (the percentage of adjacent NN intervals that differ from each other by greater than 50 ms). Long-term HRV time domain metrics were excluded in this study due to the short time frame of recording (5 min). All calculations for HRV were performed using code that we developed in Python (3.5.2) that were validated using Kubios HRV Premium (version 3.3). Our code for HRV analysis is available here: https://github.com/Big-Ideas-Lab/OptimizingWearableSR.

Metrics of both HR and HRV from ECG and downsampled PPG were compared. The differences between each downsampled PPG and the ECG were calculated and statistically evaluated using a *Bland–Altman* analysis, where mean bias and 95% limits of agreement were calculated. Paired two-sided *t*-tests were performed between the ECG and each of the downsampled PPG metrics (significance threshold is Bonferroni-corrected *p* < 0.0005, 100 different analyses performed, 10 metrics × 10 sampling rates). All statistical analyses were conducted in Python (3.8.3).

We performed simple linear regressions between each ECG metric and the error (difference between ECG metric and PPG metric) for each sampling rate to visualize whether errors are driven by higher or lower values in each metric and for each sampling rate.

### Data Volumes with Decreasing Sampling Rates

Required data storage was computed from the file size of the raw signals of the ECG and each of the downsampled PPG signals. The file size was used as an input to estimate of the amount of data storage required for a 24-h period for each of these modalities. Costs of on-premise, secure medical data storage range from $0.15 to $0.30/GB/month [3]. We performed cost calculations using Equation 1 for a user (*p*) for 12 months (*n* = 12) with an average days per month of 30.42 using our calculated data volumes over 24 h and the minimum cost $0.15/GB/month.
(1)
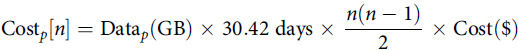



## Results

### Optimal Sampling Rate of PPG for HR

**Fig. 3.**
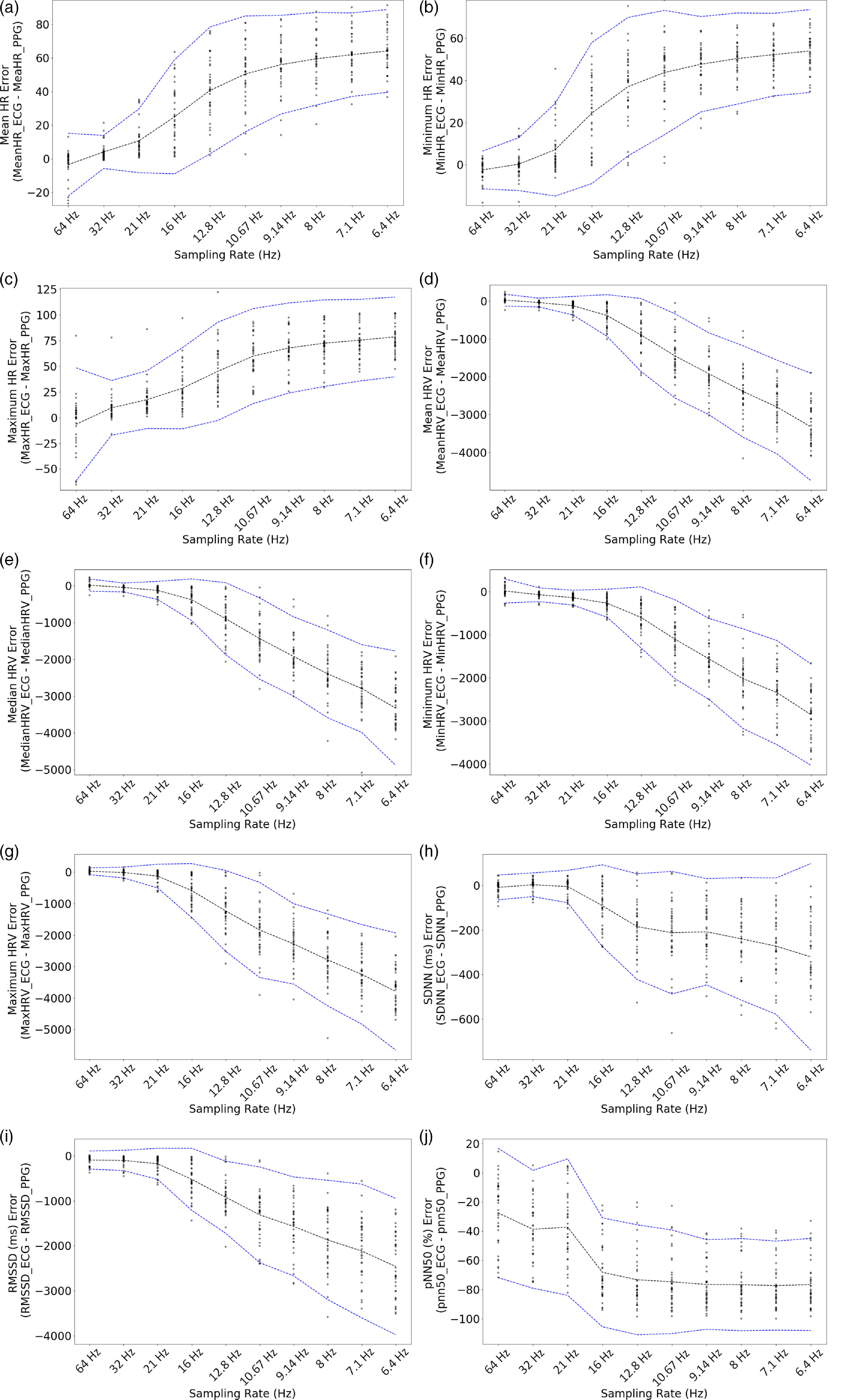
Bland–Altman analysis results between HR metrics and time domain HRV metrics according to sampling rate. (a) Mean HR, (b) minimum HR, (c) maximum HR, (d), mean HRV, (e) median HRV, (f) minimum HRV, (g) maximum HRV, (h) SDNN, (i) RMSSD, and (j) pNN50%. Blue dashed lines: limits of agreement; black dashed line: bias; points represent differences between ECG and PPG sampling rate.

The mean and standard deviation of the HR metrics for each of the downsampled signals across the 37 subjects are reported in Table 1. Error in HR metrics increases as sampling rate decreases (Table 1; Fig. 3a–c). Decreased sampling rate biases measurements toward lower HR values (Table 1; Fig. 3a–c). Bland–Altman analysis demonstrates that the limits of agreement increase as the sampling rate is decreased (Fig. 3a–c; Supplementary Table 1). There is a rise in the positive bias and confidence intervals between 21 and 16 Hz, with the greatest error occurring at the lowest sampling rate, 6.4 Hz. Paired *t*-tests between each HR metric calculated from the ECG and each sampling rate are shown in Table 1. In our sample, there was insufficient information to suggest sampling PPG at 64 Hz estimates a significantly different mean HR than ECG using paired *t*-tests. For minimum HR and maximum HR, within our sample, there was insufficient information to suggest sampling the PPG signal at 32 and 64 Hz is significantly different from the ECG using paired *t*-tests. Our linear regression visualization showed that mean HR error between ECG and PPG is not affected by lower or higher values at 64 Hz but is affected at other sampling rates, where error increases with increasing mean HR (Supplementary Fig. 1). Minimum HR error is not affected by lower or higher values at 32–64 Hz but is affected at other sampling rates, where error increases with increasing minimum HR. At all sampling rates, maximum HR error between PPG and ECG increases as maximum HR increases (Supplementary Fig. 1).

**Table 1.** HRV and HR metrics across sampling rates statistics of HR and HRV analysis across sampling rates in the time domain (mean ± standard deviation; results of paired two-sided *t*-test with Bonferroni multiple hypothesis correction

Sampling rate	Mean HR (bpm)	Minimum HR (bpm)	Maximum HR (bpm)	Mean HRV	Median HRV	Minimum HRV	Maximum HRV	RMSSD (ms)	SDNN (ms)	pNN50 (%)
ECG	79.2 ± 11.7	67.0 ± 9.1	96.1 ± 18.7	778.6 ± 122.0	779.8 ± 124.0	643.3 ± 102.0	912.8 ± 129.0	43.5 ± 20.4	53.0 ± 16.4	19.5 ± 14.9
64 Hz	82.7 ± 14.8	69.4 ± 10.5	102.7 ± 27.3	758.1 ± 148.0	759.7 ± 152.3	626.0 ± 168.2	885.2 ± 143.2	138.5 ± 101.4^*^	61.5 ± 30.7	47.0 ± 23.2^*^
32 Hz	75.2 ± 11.5^*^	66.7 ± 11.3	86.6 ± 13.6	822.0 ± 133.7^*^	822.7 ± 134.5	712.5 ± 130.0^*^	925.5 ± 154.3	147.4 ± 113.3^*^	49.7 ± 26.0	58.2 ± 19.2^*^
21 Hz	68.5 ± 11.0^*^	59.8 ± 11.0^*^	78.7 ± 11.9^*^	904.6 ± 151.6^*^	903.6 ± 153.3^*^	781.1 ± 126.2^*^	1038.9 ± 197.9	221.6 ± 169.6^*^	57.6 ± 33.8	56.8 ± 17.7^*^
16 Hz	54.2 ± 9.5^*^	42.6 ± 10.7^*^	67.6 ± 10.5^*^	1162.7 ± 213.6^*^	1157.3 ± 223.8^*^	909.2 ± 139.8^*^	1497.7 ± 357.6^*^	566.1 ± 341.5^*^	143.5 ± 87.4^*^	87.7 ± 7.0^*^
12.8 Hz	38.6 ± 8.7^*^	30.0 ± 8.5^*^	51.1 ± 11.0^*^	1677.8 ± 392.0^*^	1673.4 ± 401.2^*^	1239.3 ± 309.5^*^	2143.9 ± 547.7^*^	964.5 ± 397.0^*^	237.7 ± 114.1^*^	92.8 ± 6.9^*^
10.7 Hz	28.7 ± 6.9^*^	23.3 ± 6.9^*^	36.2 ± 8.2^*^	2230.0 ± 465.3^*^	2215.3 ± 461.3^*^	1745.5 ± 401.9^*^	2751.2 ± 667.9^*^	1355.1 ± 534.0^*^	264.7 ± 134.0^*^	94.2 ± 6.2^*^
9.1 Hz	23.2 ± 4.8^*^	19.4 ± 3.7^*^	28.4 ± 6.4^*^	2702.5 ± 472.4^*^	2702.5 ± 471.0^*^	2203.0 ± 413.5^*^	3193.6 ± 576.2^*^	1613.3 ± 556.8^*^	260.9 ± 115.8^*^	96.0 ± 4.7^*^
8 Hz	19.7 ± 3.8^*^	16.7 ± 3.0^*^	23.7 ± 6.0^*^	3168.9 ± 542.4^*^	3175.2 ± 535.3^*^	2661.3 ± 541.3^*^	3702.7 ± 669.4^*^	1913.0 ± 672.7^*^	292.4 ± 135.0^*^	96.1 ± 3.3^*^
7.1 Hz	17.3 ± 2.5^*^	14.9 ± 2.6^*^	20.8 ± 3.7^*^	3580.5 ± 581.6^*^	3575.8 ± 562.4^*^	2986.4 ± 569.4^*^	4161.3 ± 770.3^*^	2159.0 ± 757.5^*^	325.3 ± 156.3^*^	96.8 ± 3.1^*^
6.4 Hz	15.1 ± 2.2^*^	13.1 ± 2.2^*^	17.6 ± 2.8^*^	4105.8 ± 676.0^*^	4110.5 ± 746.8^*^	3498.5 ± 550.2^*^	4705.7 ± 907.9^*^	2504.0 ± 772.3^*^	373.1 ± 210.1^*^	96.0 ± 3.8^*^

* *p* < 0.0005 significant difference between ECG.

### Optimal Sampling Rate of PPG for HRV

Evaluating HRV accuracy using PPG revealed that, similar to HR, error in HRV metrics increases as PPG sampling rate decreases (Table 1; Fig. 3d–j). The mean and standard deviations of the HRV metrics (mean, median, minimum, maximum, SDNN, RMSSD, pNN50%) for each of the downsampled signals across the 37 subjects are reported in Table 1. Decreased sampling rate biases measurements toward higher HRV values. The greatest differences between the PPG-derived metrics and the ECG-derived metrics were found in RMSSD and pNN50% (Fig. 3i, j). Bland–Altman analysis demonstrates that the limits of agreement increase as the sampling rate is decreased (Fig. 3d–j; Supplementary Table 1). Again, there is a rise in the negative bias and confidence intervals between 21 and 16 Hz, with the greatest error occurring at the lowest sampling rate, 6.4 Hz. Paired *t*-tests between each HRV metric calculated from the ECG and each sampling rate are shown in Table 1. In our sample, there was insufficient information to suggest sampling PPG at 64 Hz estimates a significant difference in mean HRV and minimum HRV than ECG using paired *t*-tests. For median HRV, the paired *t*-tests showed that in our sample, there was insufficient evidence to suggest a significant difference between the ECG and the PPG signal sampled at 32 and 64 Hz. For maximum HRV and SDNN, the paired *t*-tests showed that in our sample, there was insufficient evidence to suggest a significant difference between the ECG and the PPG signal sampled at 21, 32, and 64 Hz. Because SDNN achieved accuracy across 21–64 Hz according to the paired *t*-tests and the Bland–Altman analysis, we examined its Bland–Altman analysis in more detail in the spectrum 21–64 Hz (Supplementary Fig. 2). In our sample, there was evidence to suggest a significant difference for RMSSD or pNN50 at every sampling rate tested in the study, indicating that these metrics require a sampling rate >64 Hz to achieve optimal accuracy from a wrist-worn device. The linear regression visualization showed that mean HRV error between ECG and PPG is minimally affected by higher mean HRV at 64 Hz and at all other sampling rates error increases with decreasing mean HRV (Supplementary Fig. 3). Median HRV follows the same trend as mean HRV. Minimum HRV error is only minimally affected by lower or higher values at 21–64 Hz but is affected at other sampling rates, where error increases with decreasing minimum HRV (Supplementary Fig. 3). Maximum HRV error is only minimally affected by lower or higher values at 32–64 Hz but is affected at other sampling rates, where error increases with decreasing maximum HRV (Supplementary Fig. 3). At all sampling rates, SDNN, RMSSD, and pNN50 error between PPG and ECG increases as SDNN, RMSSD, and pNN50 decrease, respectively (Supplementary Fig. 3).

### Reducing Sampling Rates Translates to Decreased Resource Requirements

**Fig. 4.**
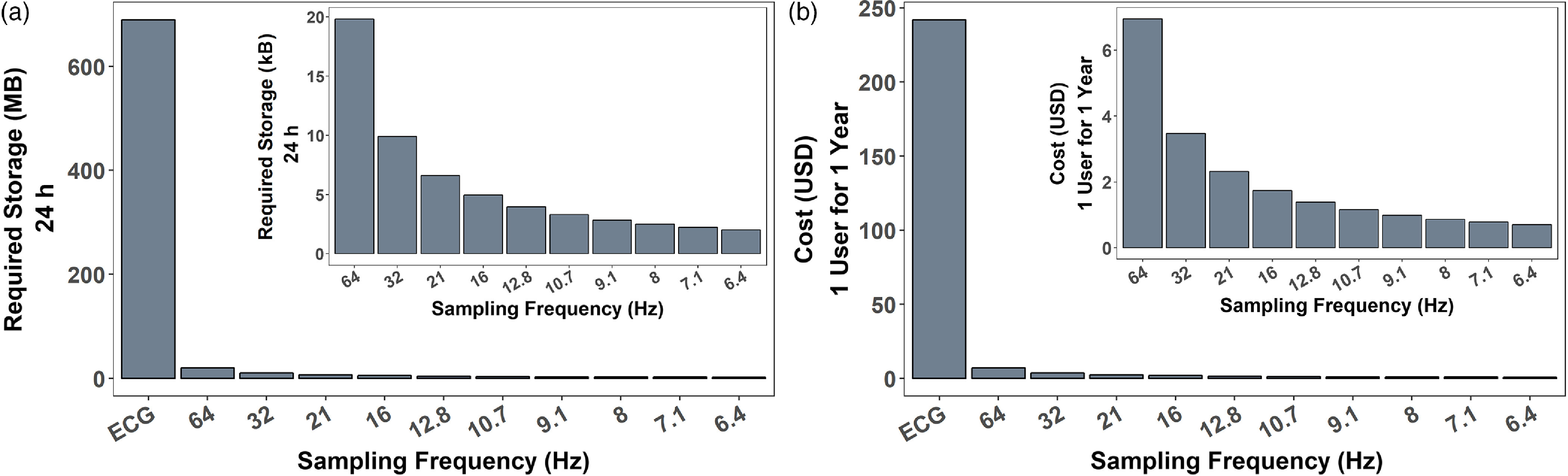
Data storage and costs of ECG compared to PPG at various sampling rates. (a) Required data storage for 24 h. (b) Cost in USD for 1 user for 1 year at the given sampling frequency.

Data storage requirements for ECG are 99.0X greater than PPG sampled at 64 Hz. PPG sampled at 32 Hz reduces the data storage requirement by an additional 50% (Fig. 4a). Costs of on-premise, secure medical data storage range from $0.15 to $0.30 per GB per month [3]. Continuous ECG data storage cost is $242.07 per user/year (Fig. 4b). PPG sampled at 64 Hz costs $6.95/user/year. Reducing the sampling rate to 32 Hz translates to reduction in cost and storage of 50% (to $3.48/user/year).

## Discussion

There are currently 60.5 million people in the United States using 117 million wearable devices. These numbers are expected to double in the next 3 years [21]. This significant rise in the accessibility of wearable devices and recent improvements in mobile health technologies provide an unprecedented opportunity to revolutionize chronic disease detection and intervention through the development of digital biomarkers, which are markers of disease extracted from digitally collected data [22]. With wearables posed to transform personalized medicine, it is critical to evaluate the limitations of wearable device sensors that will be used to make healthcare decisions [23–25].

### Framework for Assessing Optimal Sampling Rate

Continuous monitoring presents significant and unforeseen challenges to the healthcare industry. Enormous amounts of healthcare data are being generated daily. In the drive for personalized medicine, wearable sensor data will need to be stored in an accessible way. This sensor data may be used in the future for research purposes and potentially in healthcare practice by applying new digital biomarker algorithms to previously collected patient data to enable longitudinal evaluation of patients. As we have shown, sampling rates are directly proportional to data resource requirements. The ability to reduce a sampling rate by half would reduce data storage requirements and associated costs by nearly 50%. In addition, higher sampling rates have increased power consumption, which decreases battery life. Thus, another motivation to decrease sampling rate is the trade-off between battery power consumption and sampling rate.

In this study, we used optical heart rate monitors to demonstrate the connection between sampling rate and resource utilization (e.g., data storage space and cost, device battery life). We developed methodology for determining the optimal sampling rate for clinical and research relevance that minimizes the sampling rate requirements. This is extensible to other wearable sensors, including accelerometry, blood pressure, spO_2_, glucose monitors, and other traditional clinical metrics that can now be measured outside of the clinic using wearable sensors.

### Optimal Sampling Rate for HR and HRV Metrics for Wrist-Worn Wearable Devices

When considering use of optical heart rate measurements on the wrist, it is important to acknowledge that biases exist, including consistently lower HR metrics and higher HRV metrics from the PPG as compared with the ECG. Health-related insights drawn from PPG data should take these biases into account. Our results, using a combination of Bland–Altman analysis, paired *t*-tests, and linear regression visualizations, demonstrate that we can reduce the sampling rate by half (from 64 to 32 Hz) or more (from 64 to 21 Hz) in half of the HR and HRV metrics studied here (Table 1; Fig. 3). For applications that require high precision of mean HR and/or mean HRV, 64 Hz may be the optimal sampling frequency. For applications requiring high accuracy in all other metrics, a 32-Hz PPG sampling rate would maintain sufficient accuracy while reducing the storage requirements for this data by half. For applications that require less precision, sampling rates of 21 Hz may be appropriate, further reducing the data storage requirements. This study shows that, in our sample, there was insufficient information to suggest that there were significant differences between ECG and PPG sampled at 64 Hz for all HR metrics and all HRV time domain metrics with the exception of pNN50% and RMSSD, which would require a sampling rate >64 Hz to achieve optimal accuracy from a wrist-worn device. Thus, if an application requires the use of pNN50% or RMSSD, as is the case in certain psychiatric fields [26], a higher sampling rate or a more robust motion artifact removal algorithm will be necessary to achieve accuracy of the pNN50% or RMSSD value at sampling rates less than or equal to 64 Hz on a wrist-worn device. pNN50% and RMSSD have been shown to achieve accuracy in finger probe PPG at higher sampling rates [14] and further study would be necessary to determine optimal sampling rates for pNN50% and RMSSD on wrist-worn PPG. Researchers determining the minimum sampling rate for their particular application should examine the biases and loss of information presented in our analyses. There is not a “one-size-fits-all” model that will work for every application, thus it is important to examine each of the analyses presented in the context of the acceptable loss of information and bias for each particular application.

The linear regressions to visualize error between ECG and PPG at the range of metrics and sampling rates provides further support for the minimum sampling rate ranging from 21 to 64 Hz, depending on the metric. These visualizations further show that wrist-worn PPG sensor HR metrics are biased lower than the reference standard ECG metrics and that HRV metrics are biased higher than the reference standard ECG metrics, so higher values of HR metrics have higher error and lower values of HRV metrics have higher error.

Reducing data volumes by half (from 64 to 32 Hz) or more (from 64 to 21 Hz) would significantly reduce the data storage and management necessary for clinically relevant PPG sensor data (Fig. 4). This would allow data from these sensors to be stored, accessed, and utilized within the clinical framework, which would provide a more comprehensive picture of a patient’s health and wellness. It would enable the diagnosis of autonomic dysfunction and certain cardiometabolic diseases from longitudinal HR and HRV metrics. Through daily monitoring via wearable sensors, personalized medicine will be integrated into the healthcare framework through digital biomarkers, providing significant benefits to both patients and providers.

### Limitations and Future Directions

This study is limited in that it only includes heart rate and time domain HRV metrics. Frequency domain metrics were excluded from this study because there is no previous literature to support wearable PPG monitors being able to accurately determine frequency domain metrics at the highest sampling rate recorded of 64 Hz. The study size is limited to 37 participants and the short time period of data obtained (5 min) is a limitation of this study. Another study limitation is the narrow age range of subjects. Future studies with more participants or a longer duration of measurement would be useful for validation of these findings. Future studies with age ranges including older participants and children would further support the conclusions made in this study. Future studies that include analysis while a subject is in motion and participating in daily activities of living will be necessary to determine the optimal sampling rates to calculate the HR and HRV metrics in real-world conditions, including during various types and intensities of physical activity. Future studies of wrist-worn PPG sensors with original sampling rates >64 Hz may enable the analysis of the HRV metric pNN50, which appears to require sampling rate >64 Hz or a different measurement modality to achieve sufficient clinical accuracy.

## Conclusion

Including data from wearable sensors in clinical frameworks would provide a more comprehensive and longitudinal picture of a patient’s health and wellness. Continuous monitoring of relevant health parameters via wearable sensors can provide significant benefits to both patients and providers by tracking health longitudinally and monitoring responses to interventions in real time. We have used optical HR sensors as a case study for the connection between data volumes and resource requirements to develop methodology for determining the optimal sampling rate for clinical relevance that minimizes resource utilization. This is extensible to other wearable monitors, including blood pressure, spO_2_, glucose monitors, and more.

In this study, we sought to discover the optimal sampling rate for PPG data to provide clinically relevant heart rate metrics while minimizing the storage and data management requirements. This is a necessary step to facilitate rapid digitization and scalability of this wearable sensor data. We determined that the optimal sampling rate for a wrist-worn wearable device optical heart rate sensor for all HR and HRV metrics is 64 Hz (for all HRV metrics except pNN50 and RMSSD, which require sampling rate >64 Hz). For many applications that require accuracy across all metrics except mean HR, mean HRV, and minimum HRV, the PPG sampling rate can be cut in half to 32 Hz while achieving sufficient accuracy relative to the ECG. Further reduction of the sampling rate to 21 Hz may be acceptable in specific applications. We conclude that wrist-worn PPG sensor HR metrics are biased lower than the reference standard ECG metrics and that HRV metrics are biased higher than the reference standard ECG metrics. This should be considered when making physiological assessments based on PPG metrics of HR and HRV. Further studies that include analysis while a subject is in motion and participating in daily activities of living are necessary to further validate this research methodology.

## Supplementary material

For supplementary material accompanying this paper visit https://doi.org/10.1017/cts.2020.526. click here to view supplementary material
