# Supplementary material for: Optimizing sampling rate of wrist-worn optical sensors for physiologic monitoring
Source: J Clin Transl Sci. 2020 Aug 25;5(1):e34. doi: 10.1017/cts.2020.526 (PMC8057382; doi:10.1017/cts.2020.526)
Supplement: Supplementary file 1 [file S2059866120005269sup001.docx]

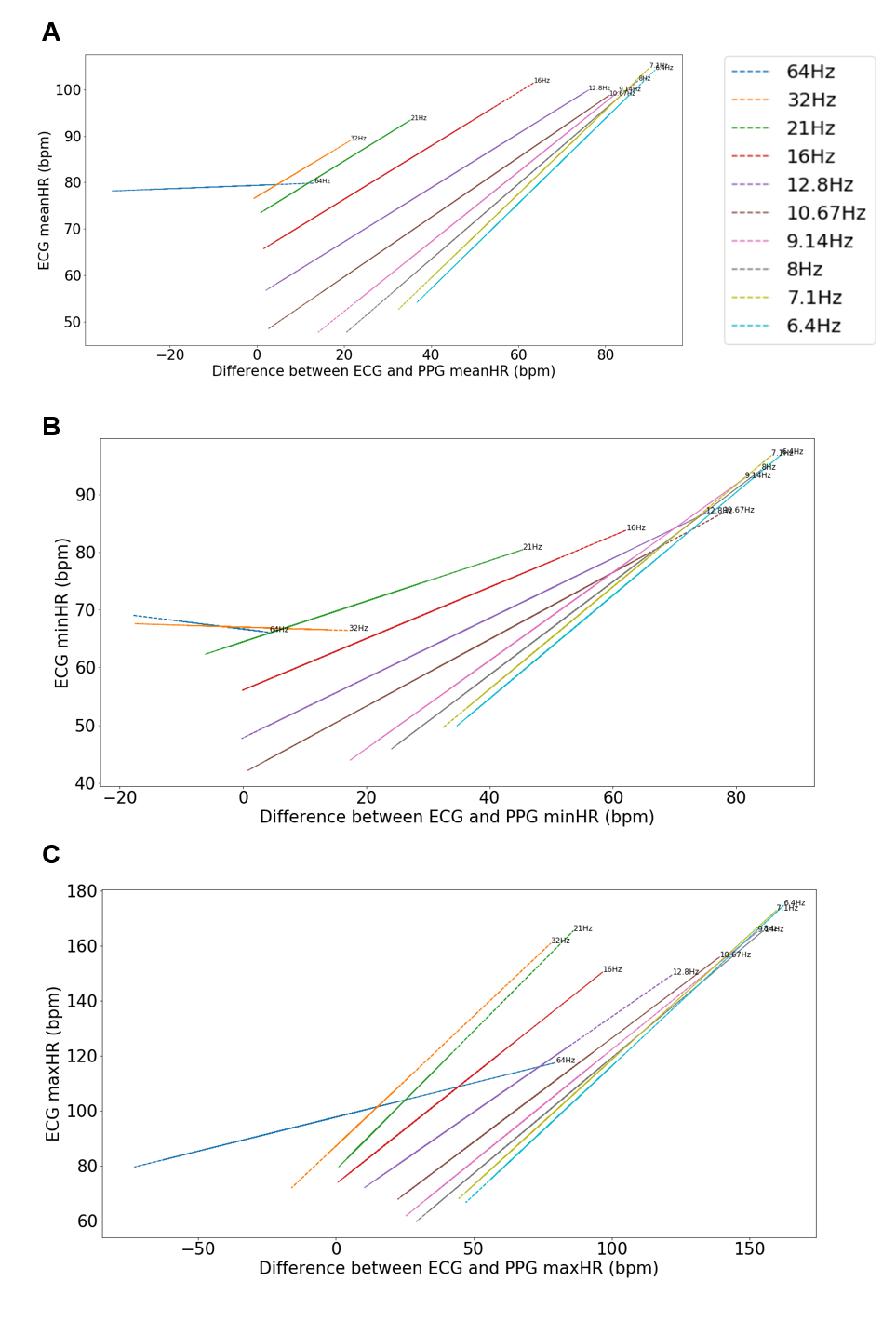


**Supplementary Figure 1.** Linear regressions between ECG metric and the difference between ECG and PPG of that metric for Mean HR (A), Minimum HR (B), and Maximum HR (C) for each sampling rate 6.4-64 Hz (shown in legend and labeled on the plot).


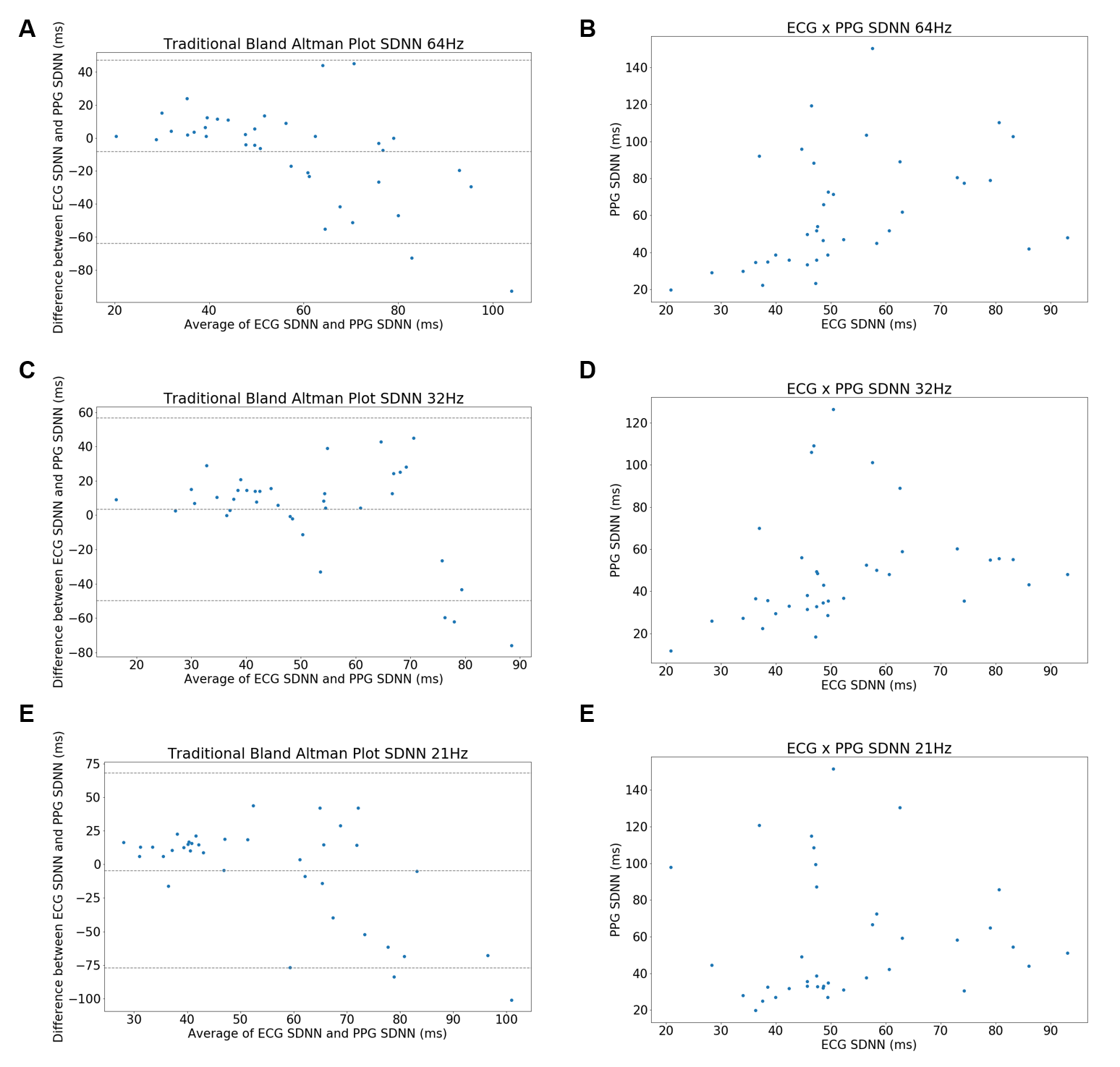


**Supplementary Figure 2.** Traditional Bland Altman plot for SDNN at 64Hz (A), 32Hz (C), and 21Hz (E). PPG SDNN by ECG SDNN at 64Hz (B), 32Hz (D), and 21Hz (F).


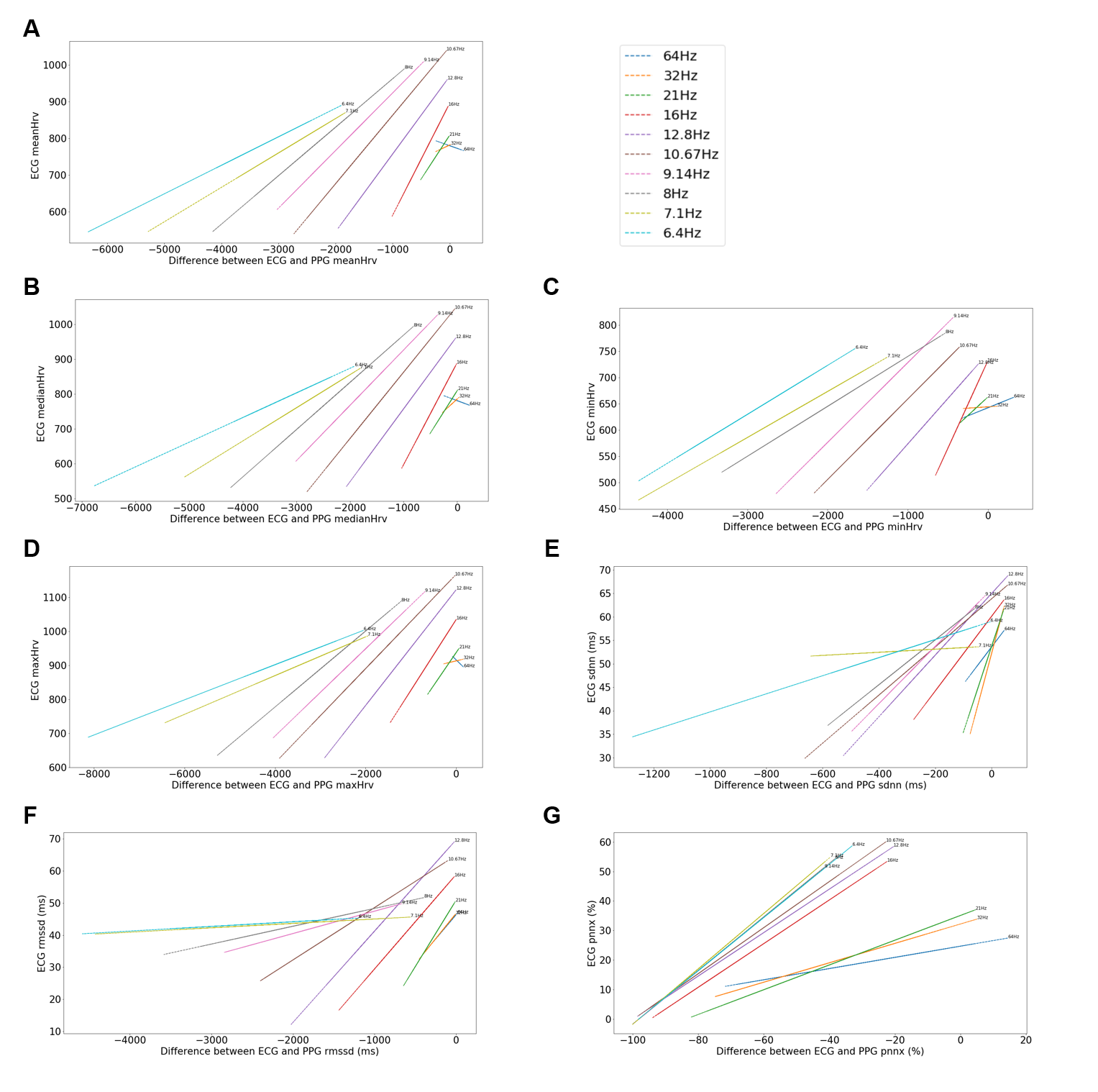


**Supplementary Figure 3.** Linear regressions between ECG metric and the difference between ECG and PPG of that metric for Mean HRV (A), Median HRV (B), Minimum HR (C), Maximum HR (D), SDNN (E), RMSSD (F), and pNN50 (G) for each sampling rate 6.4-64 Hz (shown in legend and labeled on the plot).

**Supplementary Table 1.** Bland Altman Analysis Mean Bias and LOA for each sampling rate and each HR/HRV metric.

| Sampling Rate | HR/HRV Metric | Mean Bias (Mean ± SD) | Upper LOA | Lower LOA |
| --- | --- | --- | --- | --- |
| 64Hz | Mean HR | -3.46 ± 9.52 | 15.19 | -22.11 |
| 64Hz | Minimum HR | -2.43 ± 4.55 | 6.49 | -11.35 |
| 64Hz | Maximum HR | -6.61 ± 28.08 | 48.42 | -61.64 |
| 64Hz | Mean HRV | 20.59 ± 79.48 | 176.38 | -135.19 |
| 64Hz | Median HRV | 20.16 ± 83.83 | 184.47 | -144.14 |
| 64Hz | Maximum HRV | 27.62 ± 55.33 | 136.08 | -80.83 |
| 64Hz | Minimum HRV | 17.39 ± 142.86 | 297.4 | -262.62 |
| 64Hz | SDNN | -8.46 ± 28.36 | 47.11 | -64.04 |
| 64Hz | RMSSD | -94.98 ± 102.46 | 105.85 | -295.8 |
| 64Hz | pnn50% | -27.58 ± 22.6 | 16.71 | -71.87 |
|  |  |  |  |  |
| 32Hz | Mean HR | 4.09 ± 5.01 | 13.91 | -5.73 |
| 32Hz | Minimum HR | 0.33 ± 6.4 | 12.87 | -12.2 |
| 32Hz | Maximum HR | 9.45 ± 13.59 | 36.08 | -17.18 |
| 32Hz | Mean HRV | -43.35 ± 59.1 | 72.49 | -159.19 |
| 32Hz | Median HRV | -42.89 ± 61.69 | 78.03 | -163.8 |
| 32Hz | Maximum HRV | -12.67 ± 87.52 | 158.87 | -184.2 |
| 32Hz | Minimum HRV | -69.17 ± 81.4 | 90.37 | -228.7 |
| 32Hz | SDNN | 3.33 ± 27.2 | 56.65 | -49.99 |
| 32Hz | RMSSD | -103.87 ± 115.15 | 121.83 | -329.58 |
| 32Hz | pnn50% | -38.77 ± 20.6 | 1.6 | -79.14 |
|  |  |  |  |  |
| 21Hz | Mean HR | 10.78 ± 9.71 | 29.82 | -8.26 |
| 21Hz | Minimum HR | 7.18 ± 11.19 | 29.13 | -14.76 |
| 21Hz | Maximum HR | 17.39 ± 14.35 | 45.53 | -10.74 |
| 21Hz | Mean HRV | -125.94 ± 124.31 | 117.70 | -369.59 |
| 21Hz | Median HRV | -123.81 ± 125.65 | 122.46 | -370.07 |
| 21Hz | Maximum HRV | -126.08 ± 192.09 | 250.41 | -502.56 |
| 21Hz | Minimum HRV | -137.72 ± 87.75 | 34.27 | -309.72 |
| 21Hz | SDNN | -4.63 ± 37.07 | 68.03 | -77.29 |
| 21Hz | RMSSD | -178.16 ± 175.82 | 166.45 | -522.76 |
| 21Hz | pnn50% | -37.29 ± 23.83 | 9.41 | -83.99 |
|  |  |  |  |  |
| 16Hz | Mean HR | 25.0 ± 17.31 | 58.92 | -8.92 |
| 16Hz | Minimum HR | 24.44 ± 17.01 | 57.78 | -8.91 |
| 16Hz | Maximum HR | 28.5 ± 20.14 | 67.97 | -10.98 |
| 16Hz | Mean HRV | -384.06 ± 280.75 | 166.21 | -934.33 |
| 16Hz | Median HRV | -377.52 ± 289.33 | 189.56 | -944.6 |
| 16Hz | Maximum HRV | -584.92 ± 436.64 | 270.90 | -1440.74 |
| 16Hz | Minimum HRV | -265.8 ± 165.68 | 58.93 | -590.54 |
| 16Hz | SDNN | -90.55 ± 93.59 | 92.88 | -273.99 |
| 16Hz | RMSSD | -522.58 ± 351.35 | 166.07 | -1211.23 |
| 16Hz | pnn50% | -68.22 ± 19.03 | -30.93 | -105.51 |
|  |  |  |  |  |
| 12.8Hz | Mean HR | 40.68 ± 19.24 | 78.39 | 2.96 |
| 12.8Hz | Minimum HR | 36.98 ± 16.7 | 69.72 | 4.24 |
| 12.8Hz | Maximum HR | 45.02 ± 24.43 | 92.89 | -2.86 |
| 12.8Hz | Mean HRV | -899.19 ± 490.71 | 62.59 | -1860.98 |
| 12.8Hz | Median HRV | -893.59 ± 500.11 | 86.62 | -1873.80 |
| 12.8Hz | Maximum HRV | -1231.06 ± 655.46 | 53.65 | -2515.76 |
| 12.8Hz | Minimum HRV | -595.94 ± 361.21 | 112.04 | -1303.91 |
| 12.8Hz | SDNN | -184.66 ± 121.12 | 52.74 | -422.07 |
| 12.8Hz | RMSSD | -921.05 ± 408.28 | -120.83 | -1721.27 |
| 12.8Hz | pnn50% | -73.34 ± 19.2 | -35.7 | -110.97 |
|  |  |  |  |  |
| 10.67Hz | Mean HR | 50.51 ± 17.59 | 84.98 | 16.05 |
| 10.67Hz | Minimum HR | 43.65 ± 15.01 | 73.06 | 14.24 |
| 10.67Hz | Maximum HR | 59.87 ± 23.59 | 106.11 | 13.63 |
| 10.67Hz | Mean HRV | -1451.31 ± 567.03 | -339.93 | -2562.69 |
| 10.67Hz | Median HRV | -1435.47 ± 564.43 | -329.19 | -2541.75 |
| 10.67Hz | Maximum HRV | -1838.42 ± 770.85 | -327.55 | -3349.29 |
| 10.67Hz | Minimum HRV | -1102.14 ± 466.76 | -187.29 | -2016.98 |
| 10.67Hz | SDNN | -211.75 ± 140.43 | 63.5 | -487.0 |
| 10.67Hz | RMSSD | -1311.63 ± 542.55 | -248.24 | -2375.02 |
| 10.67Hz | pnn50% | -74.74 ± 18.09 | -39.28 | -110.19 |
|  |  |  |  |  |
| 9.14Hz | Mean HR | 56.0 ± 14.97 | 85.34 | 26.65 |
| 9.14Hz | Minimum HR | 47.56 ± 11.51 | 70.12 | 25.01 |
| 9.14Hz | Maximum HR | 67.71 ± 22.38 | 111.57 | 23.84 |
| 9.14Hz | Mean HRV | -1923.9 ± 550.88 | -844.17 | -3003.62 |
| 9.14Hz | Median HRV | -1922.7 ± 550.06 | -844.57 | -3000.82 |
| 9.14Hz | Maximum HRV | -2280.77 ± 651.42 | -1003.99 | -3557.55 |
| 9.14Hz | Minimum HRV | -1559.62 ± 480.06 | -618.69 | -2500.55 |
| 9.14Hz | SDNN | -207.88 ± 121.89 | 31.02 | -446.78 |
| 9.14Hz | RMSSD | -1569.86 ± 560.38 | -471.51 | -2668.21 |
| 9.14Hz | pnn50% | -76.56 ± 15.66 | -45.87 | -107.26 |
|  |  |  |  |  |
| 8Hz | Mean HR | 59.5 ± 14.04 | 87.02 | 31.99 |
| 8Hz | Minimum HR | 50.25 ± 11.0 | 71.82 | 28.69 |
| 8Hz | Maximum HR | 72.41 ± 21.51 | 114.57 | 30.26 |
| 8Hz | Mean HRV | -2390.23 ± 616.09 | -1182.70 | -3597.77 |
| 8Hz | Median HRV | -2395.39 ± 609.97 | -1199.86 | -3590.92 |
| 8Hz | Maximum HRV | -2789.91 ± 745.41 | -1328.91 | -4250.91 |
| 8Hz | Minimum HRV | -2017.97 ± 590.56 | -860.47 | -3175.46 |
| 8Hz | SDNN | -239.42 ± 140.42 | 35.8 | -514.64 |
| 8Hz | RMSSD | -1869.55 ± 676.15 | -544.29 | -3194.8 |
| 8Hz | pnn50% | -76.69 ± 16.1 | -45.13 | -108.24 |
|  |  |  |  |  |
| 7.1Hz | Mean HR | 61.93 ± 12.68 | 86.78 | 37.07 |
| 7.1Hz | Minimum HR | 52.11 ± 9.96 | 71.63 | 32.59 |
| 7.1Hz | Maximum HR | 75.32 ± 20.3 | 115.10 | 35.54 |
| 7.1Hz | Mean HRV | -2801.9 ± 630.87 | -1565.40 | -4038.40 |
| 7.1Hz | Median HRV | -2796.02 ± 609.53 | -1601.34 | -3990.69 |
| 7.1Hz | Maximum HRV | -3248.52 ± 806.85 | -1667.10 | -4829.94 |
| 7.1Hz | Minimum HRV | -2343.04 ± 616.79 | -1134.14 | -3551.94 |
| 7.1Hz | SDNN | -272.26 ± 156.18 | 33.85 | -578.37 |
| 7.1Hz | RMSSD | -2115.48 ± 758.26 | -629.30 | -3601.67 |
| 7.1Hz | pnn50% | -77.36 ± 15.55 | -46.87 | -107.84 |
|  |  |  |  |  |
| 6.4Hz | Mean HR | 64.15 ± 12.56 | 88.77 | 39.53 |
| 6.4Hz | Minimum HR | 53.85 ± 9.99 | 73.44 | 34.26 |
| 6.4Hz | Maximum HR | 78.51 ± 19.82 | 117.36 | 39.66 |
| 6.4Hz | Mean HRV | -3327.1 ± 723.4 | -1909.23 | -4744.97 |
| 6.4Hz | Median HRV | -3330.72 ± 794.75 | -1773.0 | -4888.43 |
| 6.4Hz | Maximum HRV | -3792.86 ± 949.17 | -1932.50 | -5653.23 |
| 6.4Hz | Minimum HRV | -2855.17 ± 599.44 | -1680.28 | -4030.07 |
| 6.4Hz | SDNN | -320.27 | 98.46 | -739.01 |
| 6.4Hz | RMSSD | -2460.55 ± 773.11 | -945.24 | -3975.85 |
| 6.4Hz | pnn50% | -76.56 ± 16.1 | -45.0 | -108.12 |
